# Hemopericardium: A Comprehensive Clinical Review of Etiology and Diagnosis

**DOI:** 10.7759/cureus.52677

**Published:** 2024-01-21

**Authors:** Pawel Borkowski, Natalia Borkowska, Natalia Nazarenko, Shaunak Mangeshkar, Harriet S Akunor

**Affiliations:** 1 Internal Medicine, Albert Einstein College of Medicine, Jacobi Medical Center, New York, USA; 2 Pediatrics, SPZOZ (Samodzielny Publiczny Zakład Opieki Zdrowotnej), Krotoszyn, POL

**Keywords:** hemorrhagic cardiac tamponade, internal medicine and cardiology, cardiac tamponade, hemorrhagic pericardial effusion, pericardial effusion

## Abstract

Hemorrhagic pericardial effusion (HPE) is a subtype of pericardial effusion marked by the accumulation of serosanguineous or bloody fluid within the pericardial cavity. We present a case of a 65-year-old female who presented to the hospital with abdominal pain and was found to have pericardial effusion. The patient’s condition evolved into cardiac tamponade, and employing diagnostic techniques such as imaging and pericardiocentesis, a substantial bloody effusion was uncovered, indicative of HPE. This report underscores the complexity of HPE diagnosis and examines the non-iatrogenic etiological factors contributing to HPE, focusing on three primary causes: malignancy, infection, and autoimmune disorders. It offers a detailed exploration of each etiology, backed by current medical literature and case studies. It outlines the diagnostic strategies pertinent to each cause, underscoring the need for a tailored approach to manage such cases. It emphasizes the importance of a meticulous and individualized diagnostic process, vital for accurate identification and effective management of this condition.

## Introduction

A pericardial effusion is characterized by the accumulation of excess fluid within the pericardial sac that surpasses the normal physiological range of 15-50 mL [1]. There are different classifications of pericardial effusion, i.e. based on its onset (acute, <1 week; subacute, one week to three months; chronic, >3 months), the size on echocardiogram (mild, <10 mm; moderate 10-20 mm; large, >20 mm), and composition of the fluid (i.e. transudate (hydropericardium), chyle (chylopericardium), pus (pyopericardium), air (pneumopericardium), and blood (hemopericardium)). The occurrence of pericardial effusion is shown to rise with advancing age. In patients aged over 80 years, the rate is about 15%, compared to less than 1% in people aged 20-30 years [2].

Fluid accumulation in the pericardial sac elevates pressure, compressing the heart, predominantly the right side, due to its thinner walls [3]. This impairs the right heart’s diastolic filling, leading to underfilling of the left heart. Consequently, diminished left ventricular diastolic filling reduces stroke volume, progressively decreasing cardiac output, culminating in critical cardiovascular compromise, and finally, cardiac tamponade, classically described as Beck's triad (hypotension, jugular venous distension, muffled heart sounds). The range of pericardial effusions spans from mild, symptom-free cases to severe conditions leading to cardiac tamponade.

Echocardiography is the most reliable method for confirming the existence and quantifying pericardial effusion volume [3]. The diagnostic and treatment strategies are determined based on factors such as hemodynamics, size, the presence of pericarditis, coexisting medical conditions, and the underlying etiology. Certain diagnostic tests can be tailored for specific types of pericardial effusion, contingent upon the fluid's composition. This report focuses explicitly on hemopericardium and aims to enhance clinicians' awareness of hemorrhagic pericardial effusion (HPE), summarize the most common etiologies, and offer comprehensive diagnostic strategies for this clinical entity.

## Case presentation

A 65-year-old female with a history of hyperlipidemia presented to the Emergency Department (ED) with progressive epigastric pain radiating to the retrosternal area, aggravated by leaning forward and body movements, with no alleviating factors. Her vital signs were stable: temperature 36.6°C, blood pressure 141/83 mmHg, pulse 86 bpm, respiratory rate 15 breaths/minute. She reported no nausea, vomiting, or diarrhoea. Examination showed a soft, non-tender abdomen, elevated jugular venous pressure (12 cm), and warm skin.

**Figure 1:**
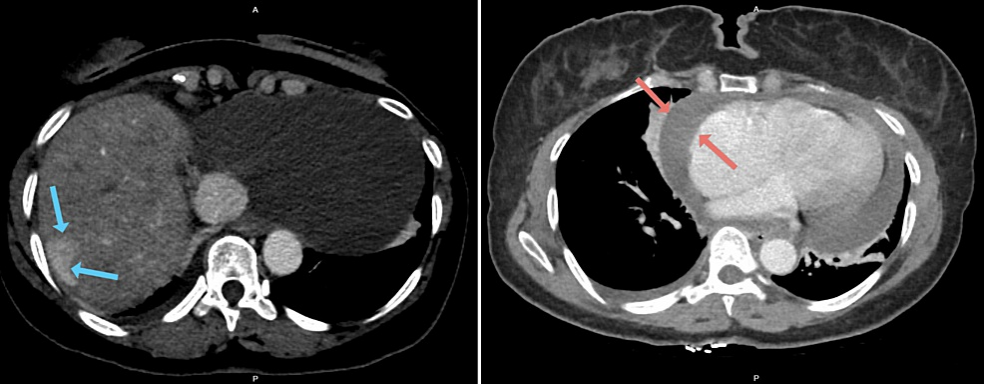
CT abdomen showing heterogeneous liver with multiple hyperenhancing foci (blue arrows) and large pericardial effusion (red arrows).

**Figure 2:**
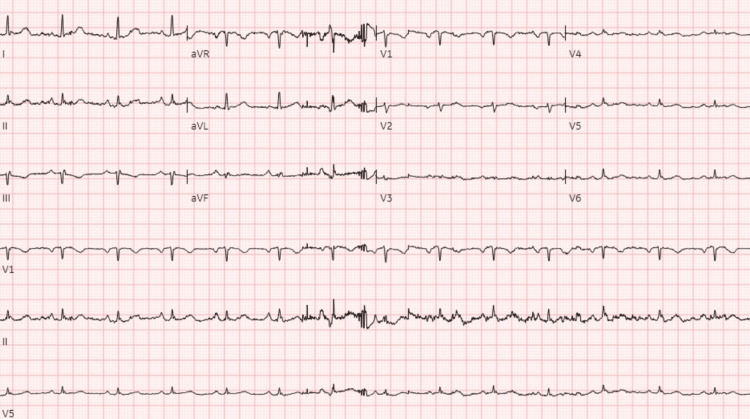
EKG showing sinus rhythm with 83 bpm, normal axis, possible left atrial enlargement in V1, and low QRS voltage in precordial leads.

Laboratory results (Table 1) showed normal levels of WBC, hemoglobin, platelets, lipase, and troponin and normal kidney function. Liver function tests revealed increased alanine transaminase (ALT) and aspartate aminotransferase (AST). The results of the respiratory viral panel were negative. CT abdomen showed heterogeneous liver with multiple hyperenhancing foci, and large pericardial effusion (Figure 1). EKG showed low QRS voltage in precordial leads (Figure 2). Further workup (Table 1) revealed slightly elevated CRP with normal erythrocyte sedimentation rate (ESR), negative interferon-gamma release assay (IGRA), HIV, hepatitis B and C screening, normal thyroid stimulating hormone (TSH), positive rheumatoid factor with normal C3 and C4, normal coagulation times, and negative cancer markers (CEA), CA19-9, CA125, alpha-fetoprotein (AFP).

**Table 1:** Laboratory results.

Laboratory Test	Actual Result	Normal Range
White blood cells (WBC)	5.49 K/uL	3.8 – 10.5 K/uL
Neutrophils (absolute)	3.3 K/uL	1.8 – 7.4 K/uL
Lymphocytes (absolute)	1.67 K/uL	1 – 3.3 K/uL
Hemoglobin	11.6 g/dL	11.5 – 15.5 g/dL
Platelets	299 K/uL	150 – 400 K/uL
Creatinine	0.7 mg/dL	0.5 – 1.3 mg/dL
Blood Urea Nitrogen (BUN)	23 mg/dL	22 – 31 mg/dL
Alanine Aminotransferase (ALT)	216 U/L	10 – 45 U/L
Aspartate Transferase (AST)	195 U/L	10 – 40 U/L
Lipase	27 U/L	7 – 60 U/L
Troponin	<0.01 ug/L	0.000 – 0.09 ug/L
C-reactive Protein (CRP)	6.5 mg/L	0 – 5 mg/L
Procalcitonin (PCT)	0.06 ng/mL	0.02 – 0.08 ng/mL
Erythrocyte Sedimentation Rate (ESR)	15 mm/hr	0 – 30 mm/hr
Interferon-γ Release Assay (IGRA)	Negative	Negative
HIV-1/HIV-2 Antigen/Antibody	Nonreactive	Nonreactive
Hepatitis B Surface Antigen	Nonreactive	Nonreactive
Hepatitis B Surface Antibody	3.5 mIU/mL (Nonreactive)	Cutoff for reactive result corresponds to 10 IU/mL
Hepatitis B Core Antibody (total)	Nonreactive	Nonreactive
Hepatitis C Antibody (total)	Nonreactive	Nonreactive
Thyroid-Stimulating Hormone (TSH)	0.9 uIU/mL	0.27 – 4.2 uIU/mL
Rheumatoid Factor (RF)	128 IU/mL	0 – 20 IU/mL
Complement 3 (C3)	133.6 mg/dL	90 – 180 mg/dL
Complement 4 (C4)	14.7 mg/dL	10 – 40 mg/dL
Prothrombin Time (PT)	12.5 seconds	9.4 – 12.5 seconds
Activated Partial Thromboplastin Clotting Time (aPTT)	28.5 seconds	25.1 – 36.5 seconds
Carcinoembryonic Antigen (CEA)	1 ng/mL	0.2 – 5 ng/mL
Cancer Antigen 19-9 (CA19-9)	25 U/mL	≤ 35 U/mL
Cancer Antigen 125 (CA125)	18 U/mL	≤ 38 U/mL
Alpha Fetoprotein (AFP)	3.3 ug/L	0 – 9 ug/L

**Figure 3:**
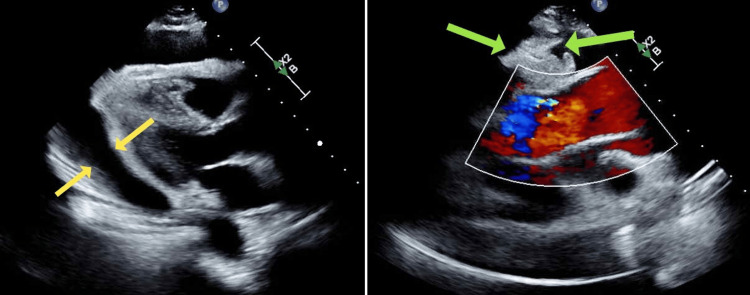
Transthoracic echocardiogram showing large, free-flowing pericardial effusion (yellow arrows) with mobile soft-tissue masses in the effusion on the visceral pericardium (green arrows).

During the hospital course, the patient developed symptomatic hypotension, tachycardia, and altered mental status. A transthoracic echocardiogram (TTE) showed a large, free-flowing pericardial effusion with mobile soft-tissue masses in the effusion on the visceral pericardium (Figure 3). She underwent urgent pericardiocentesis in the cardiac care unit, yielding 580 mL of bloody fluid. Fluid analysis showed RBC of 2,391,000 /uL, WBC of 7476 /uL (polymorphonuclear neutrophils (PMNs) 14.5%), protein of 5.4 g/dL, lactate dehydrogenase (LDH) of 976 U/L. Cytology showed lymphocytes in varied sizes with reactive mesothelial cells, RBCs, and macrophages in the background. Gram stain and cultures of the pericardial fluid did not reveal any infection. Repeat TTE showed a small pericardial effusion, the drain was removed, and the patient was transferred to the general medicine floor for further investigations, including malignancy screening and autoimmune workup. However, the patient opted to leave the hospital against medical advice before completion of the work-up and was subsequently lost to follow-up. When the patient left the hospital, the most likely diagnoses for HPE were malignancy, autoimmune disease, and viral infection.

## Discussion

Determining the underlying cause of HPE is a complex and multifaceted challenge. Invasive cardiac procedures are the most frequent cause, accounting for approximately 31% of hemopericardium cases [4]. However, a spectrum of other factors contributes to this condition. These include thoracic trauma, complications of ischemic heart disease (e.g. left ventricle free wall rupture), ascending aortic dissection which extends into the pericardium, malignancy, infection, autoimmune conditions, anticoagulants, chronic kidney disease, and medications.

Malignant pericardial effusions, which have the grimmest prognosis, are more commonly associated with non-cardiac malignancies (most widely lung cancer followed by breast cancer) than primary cardiac tumors [5,6]. Other cancer types such as lymphoma, melanoma, mesothelioma, and renal cell carcinoma are also documented but less frequent. Viral infections, notably coxsackie B virus and coronavirus disease 2019, along with bacterial infections caused by *Mycobacterium tuberculosis*, *Chlamydophila pneumoniae*, and *Staphylococcus aureus*, represent the predominant infectious etiologies of HPE described in contemporary medical literature [7-11].

Although tuberculosis-related cases have declined in the industrialized West, their prevalence remains significant among AIDS patients and in developing countries. Fungal (e.g., *Histoplasma capsulatum*) and parasitic infections (e.g., *Wuchereria bancrofti*) are also potential etiological factors but are less common [12,13]. Autoimmune conditions like rheumatoid arthritis and systemic lupus erythematosus are linked with HPE in the medical literature [14,15]. HPE is seldom the initial manifestation of autoimmune diseases, and the absence of associated symptoms like skin, joint, or mucosal signs or involvement of other extracardiac organs should not exclude the consideration of an autoimmune etiology. Notably, HPE is a recognized side effect of anticoagulants (e.g. apixaban). It is reported in association with diverse medication classes, including tumor necrosis factor-alpha (TNF-alpha) inhibitors (e.g. infliximab) and immune checkpoint inhibitors (e.g. pembrolizumab) [16-18]. In the context of end-stage renal disease, hemorrhagic pericarditis is a rare but significant complication associated with increased morbidity, representing about 3-5% of total uremic pericarditis cases [19]. Despite extensive diagnostic efforts, the specific causes of HPE can sometimes remain elusive, resulting in a classification of "idiopathic" HPE. This is compounded by the limited data on the prevalence and distribution of the various etiologies of HPE, underscoring the need for more comprehensive research in this area.

Conducting a thorough evaluation for potential causes of pericardial effusions is critical, given their tendency to recur and the risk of progressing to cardiac tamponade. An extensive diagnostic approach is required for HPE, tailored to its three most diverse etiologies. In cases of malignancy, essential investigations include pericardial fluid analysis for cytology and biomarkers such as CEA, CA19-9, CA 72-4, CA 15-3, CA125, CYFRA 21-1, AFP, and adenosine deaminase (ADA). Pericardial biopsy and a comprehensive cancer work-up, including CT of the chest, colonoscopy, mammogram, and breast ultrasound are also advised. For infections, the diagnostic process includes a range of tests like Gram stain, acid-fast bacillus (AFB) stain, cultures for bacterial (including mycobacteria) and fungal pathogens, assays for interferon-gamma or lysozyme, and HIV screening. Polymerase chain reaction (PCR) for specific infectious agents is recommended in cases where management decisions are influenced by the findings, such as detecting cytomegalovirus in transplant patients [20]. However, the efficacy of PCR in providing definitive results can be limited. A parasitic work-up is suggested after excluding other causes due to its rarity. In autoimmune conditions, initial testing should include antinuclear antibody (ANA), anti-doubles stranded (ds)DNA, anti-Smith, complements C3 and C4, rheumatoid factor, and anti-cyclic citrullinated peptide. If results are inconclusive, a broader panel is recommended, encompassing anti-Sjögren's-syndrome (SS)A, anti-SSB, anti-CREST (calcinosis, Raynaud phenomenon, esophageal dysmotility, sclerodactyly, and telangiectasia), anti-Scl70, anti-ribonucleoprotein (RNP), anti-PL-7, anti-PL-12, perinuclear anti-neutrophil cytoplasmic antibodies (p-ANCA), cytoplasmic ANCA (cANCA), anti-hepatitis C virus (HCV), and myositis panels.

The prognosis and treatment strategy for HPE are heavily influenced by its underlying cause, underscoring the importance of accurately identifying the specific etiology to facilitate targeted therapeutic interventions. The rarity of hemopericardium cases means that most current knowledge is sourced from case reports and limited-scale retrospective studies. Enhanced research efforts in this domain are crucial for deepening our understanding of hemopericardium, a step that is essential for elevating the quality of care and outcomes for individuals diagnosed with this condition.

## Conclusions

The causes of HPE include invasive cardiac procedures, thoracic trauma, complications of ischemic heart disease (e.g. left ventricle-free wall rupture), malignancy, infection, autoimmune conditions, anticoagulants, chronic kidney disease, and medications. The diverse causes of HPE, particularly malignancy, infection, and autoimmune disorders, demand an exhaustive diagnostic work-up to ensure accurate identification and appropriate treatment. Furthermore, due to the diverse and often elusive origins of HPE, there is a notable lack of epidemiological data. This gap underscores the necessity for more comprehensive clinical studies and data gathering to deepen our understanding of this clinical entity.
